# Quantitative investigation of drug-drug interaction between bergenin and vilazodone in rats through UPLC-MS/MS assay

**DOI:** 10.1186/s13065-024-01203-5

**Published:** 2024-05-03

**Authors:** Mengming Xia, Xinhao Xu, Chaojie Chen, Hualu Wu, Ren-ai Xu, Changlv Wang

**Affiliations:** 1 The Affiliated LiHuiLi Hospital of Ningbo University, Ningbo, Zhejiang China; 2 The First Affiliated Hospital of Wenzhou Medical University, Wenzhou, Zhejiang China

**Keywords:** Vilazodone, M10, Pharmacokinetics, Bergenin, UPLC-MS/MS

## Abstract

In this study, we firstly established and verified a method by ultra performance liquid chromatography tandem mass spectrometry (UPLC-MS/MS) for the analysis of vilazodone and its metabolite M10 in rat plasma, then this method was used to explore the pharmacokinetics of vilazodone and M10 present or absence of 80 mg/kg bergenin in rats. Protein precipitation with acetonitrile was used to prepare the samples in this research. The mobile phase for liquid chromatography was consisted of 0.1% formic acid aqueous solution and acetonitrile. Brexpiprazole was used as the internal standard (IS), and the multiple reaction monitoring (MRM) mode was used for detection. The verification items required by the US Food and Drug Administration (FDA) guidelines such as selectivity, sensitivity, linearity, stability, recovery and matrix effect of this method were all met the standards. Besides, rats were used to explore the drug-drug interaction between vilazodone and bergenin, which were divided into two groups, and separately gavaged with the same-volume of carboxymethyl cellulose sodium (CMC-Na) solution and 80 mg/kg bergenin, respectively. The results showed that bergenin significantly affected the metabolism of vilazodone. It suggested that there was a potential drug-drug interaction between bergenin and vilazodone in rats. In clinical application, we should pay attention to the dose of vilazodone when in combination with bergenin.

## Introduction

Two species of the Genus Bergenia, B. crassifolia and B. purpurascens are commonly used as traditional Chinese medicines, and one of their main active ingredients is bergenin [1, 2]. Studies have shown that bergenin widely exists in the Genus Bergenia [3, 4]. And, it has been proven to have multiple pharmacological functions such as antioxidant, antimicrobial, anti-inflammatory, neuroprotective effect [5–8], and also can be used in the treatment of various diseases, including diabetic nephropathy, asthma and Alzheimer’s disease [7, 9, 10]. Bergenin is also commonly found in some cosmetics in daily life. In addition, bergenin is a natural peroxisome proliferator-activated receptor-γ (PPAR-γ) agonist and a human liver cytochrome P450 (CYP) 3A4 and 2C9 inhibitor, suggesting a possible drug-drug interaction of bergenin with co-administered drugs [11]. Therefore, further researches for the potential drug-drug interaction are necessary to be explored.

**Fig. 1:**
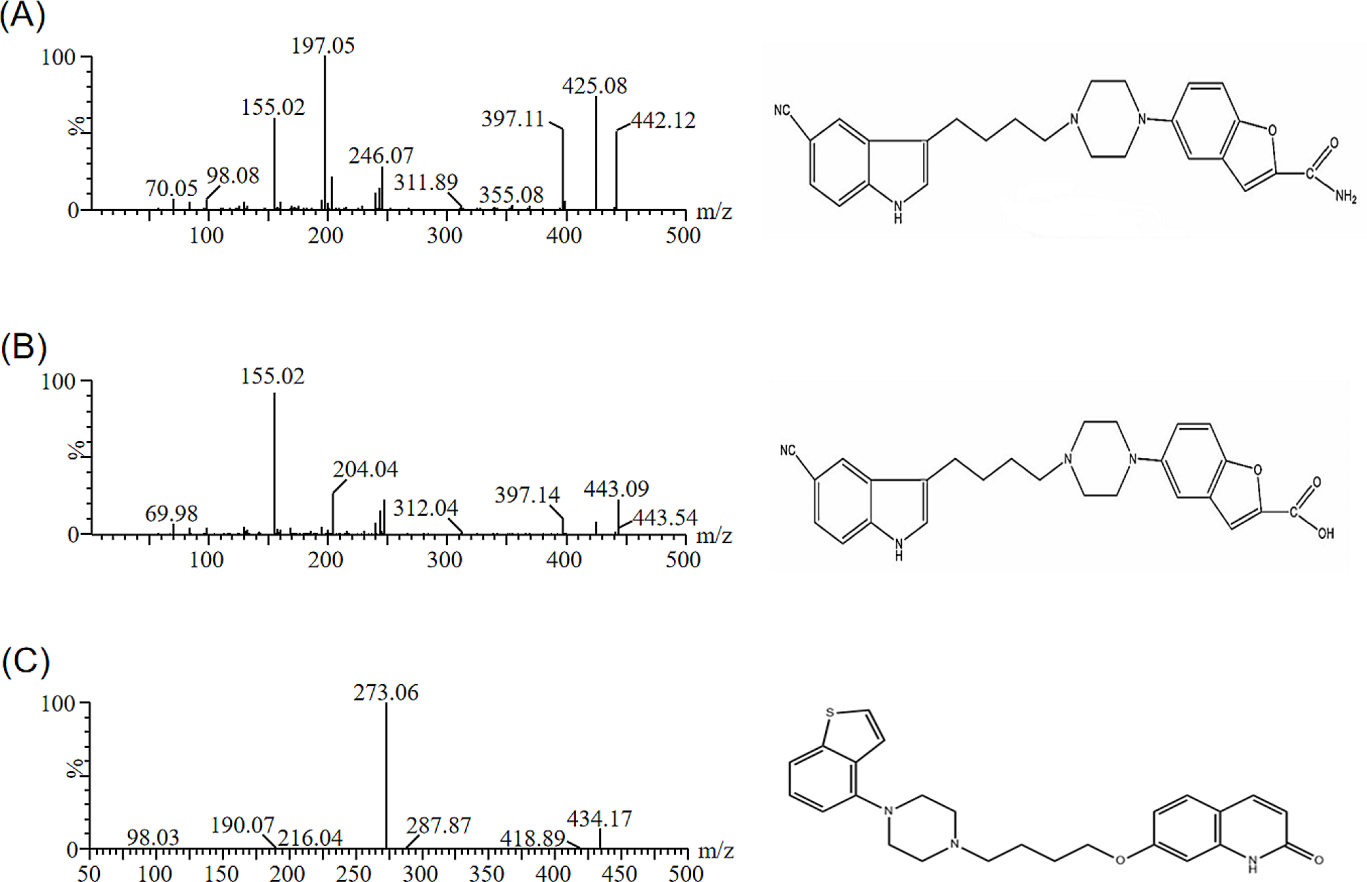
Product ion mass spectra and chemical structure of vilazodone **(A)**, M10 **(B)**, and IS **(C)**

Vilazodone (Fig. 1A), a 5 hydroxytryptamine receptor 1A (5-HT1A) partial agonist and selective reuptake inhibitor, has been approved by the FDA for the treatment of major depressive disorder in adults [12, 13]. Previous studies have shown that vilazodone is also effective in the treatment of generalized anxiety disorder and adult separation anxiety disorder [14, 15]. Animal studies suggest vilazodone may have potential antidyskinetic efficacy in Parkinson’s disease patients [16]. Vilazodone is metabolized mainly by the hepatic CYP3A4 enzyme system and one of the major inactive metabolites of vilazodone is M10 (Fig. 1B) [17]. Considering the wide clinical application of bergenin and its CYP3A4 inhibitor properties, there may be potential bergenin intake in patients who take vilazodone on a daily basis. Therefore, the potential drug-drug interaction between bergenin and vilazodone needs to be explored.

Previous studies have reported the use of liquid chromatography tandem mass spectrometry (LC-MS/MS) method to quantitatively detect the concentration of vilazodone in human [18], dog [19], and rat plasma [20]. Only one report has simultaneously determined human plasma concentrations of vilazodone and its major metabolite by HPLC-DAD and HPLC-MS [21]. However, this study used long analytical time and complicated solid-phase extraction procedure, and did not further explore the application of the method in pharmacokinetics. Besides, this chromatography method may not meet the high sample throughput requirements in complex biological analysis, and cannot be applied to drug-drug interaction studies. Thus, an efficient quantitative ultra performance liquid chromatography tandem mass spectrometry (UPLC-MS/MS) method developed for the simultaneous determination of vilazodone and M10 in rat plasma is necessary and application of this method in drug-drug interaction studies needs to be validated.

In this study, we established a stable, rapid, and precise measurement method to simultaneously detect the concentrations of vilazodone and M10 in rat plasma by UPLC-MS/MS. In addition, we applied this method to the pharmacokinetic study of vilazodone and explored the drug-drug interaction between bergenin and vilazodone in rats.

## Materials and methods

### Chemicals

The formic acid and the standard reagents including vilazodone, M10 and brexpiprazole (used as the internal standard, IS, Fig. 1C), were all purchased from Beijing Sunflower and Technology Development CO., LTD (Beijing, China). All reagents were analytical grade, and the purities of standard reagents were > 98%. Acetonitrile and methanol were both HPLC grade, and were purchased from Merck Company (Darmstadt, Germany). Deionized water used in the experiment was purified by the Milli-Q water purification system (Millipore, Bedford, USA).

### Instrumentations and UPLC-MS/MS conditions

The UPLC-MS/MS system (Milford, MA, USA) was consisted of a Waters Xevo TQ-S triple quadrupole tandem mass spectrometer with an electro-spray ionization (ESI) source and a Waters ACQUITY UPLC I-Class system with Acquity UPLC BEH C18 column (2.1 mm × 50 mm, 1.7 μm). The column temperature was set at 40 °C. Masslynx 4.1 software equipped with the system was used for instrument control and data acquisition.

The compositions of gradient elution solvent were acetonitrile (A) and 0.1% formic acid in water (B). The injection volume was 3.0 µL, and the injection temperature was 10 °C. The gradient program was as follows: 90% B for 0.5 min, 90% decreased to 10% B in 0.5 min, 10% B for 0.4 min, 10% increased to 90% B in 0.1 min, 90% B for 0.5 min. The flow rate was 0.30 mL/min, and the run time was 2.0 min for total.

Mass spectrometry was performed in positive ion mode, and the measurements of the levels of the two targets and IS were accomplished using multiple reaction monitoring (MRM). The fixed parameters were as follows: collision gas 0.20 mL/min, capillary voltage 2.0 kV, cone gas 150 L/h, desolvation gas 1000 L/h, desolvation temperature 600 °C. Quantitative detection of ion transitions for vilazodone, M10 and IS were *m/z* 442.12→197.05, *m/z* 443.09→155.02, and *m/z* 434.17→273.06, respectively. The specific mass spectrometric parameters were shown in Table 1.

**Table 1:** Specific mass spectrometric parameters and retention times (RTs) for vilazodone, its metabolite and IS, including cone voltage (CV), and collision energy (CE)

Analytes	Precursor ion	Product ion	CV (V)	CE (eV)	RT (min)
Vilazodone	442.12	197.05	30	30	1.38
M10	443.09	155.02	30	40	1.39
IS	434.17	273.06	30	25	1.42

### Calibration standards and quality control (QC)

Methanol was used to separately prepare 1.0 mg/mL solutions of the IS, vilazodone and its metabolite as stock solutions. The stock solutions were further diluted with methanol to prepare the working solutions of calibration standard for the regents of different concentrations. 100 ng/mL was set to the concentration of the IS working solution. The concentration of the calibration curve was sequentially set as 1, 2, 5, 10, 20, 50, 100, 200 ng/mL. The concentration of the QC samples was sequentially set as 1, 2, 40, 180 ng/mL. All the above solutions and samples were stored at 4 °C in freezers for further analysis.

### Plasma preparation

Protein precipitation, using acetonitrile as the precipitant, was used in the preparation of plasma samples in this study. First, 20 µL of IS working solution and 300 µL acetonitrile as a precipitant were sequentially added into 100 µL of plasma. Vortex was performed to mix the solution after each adding. Then, supernatant (100 µL) was obtained after centrifugating it at 13,000 × rpm for 10 min, and 3.0 µL of it was used for the quantitative analysis at last.

### Animal experiments

The Institutional Ethics Committee of The First Affiliated Hospital of Wenzhou Medical University (Zhejiang, China) approved this animal experiment, which strictly adhered to the National Institute of Health (NIH) guidelines. The Sprague-Dawley (SD) rats weighing 200 ± 20 g were purchased from the Laboratory Animal Center of The First Affiliated Hospital of Wenzhou Medical University (Zhejiang, China). All rats were kept in proper environment without restriction of food and water for 7 days. Then, they were fasted for 12 h before the experiment and randomly divided into two group A and group B. The rats in group B and group A were orally administrated with 80 mg/kg of bergenin and the equal volume of carboxymethyl cellulose sodium (CMC-Na) solution, respectively. 4 mg/kg vilazodone as a single dose was oral administrated for all rats after 0.5 h. The time points of caudal veins blood collection were set as 0.333, 0.667, 1, 1.5, 2, 3, 4, 6, 8, 12, 24 and 48 h after administration. Plasma samples were immediately centrifugated for 8 min at 4000 ×g and stored in -80 °C refrigerator until analysis.

Euthanasia of experimental animals was performed using the anesthesia method according to the AVMA Guidelines for the Euthanasia of Animals. After completion of the experiment, all experimental animals were euthanized by intravenous pentobarbital (150 mg/kg). After ensuring that the animals were free of life pointers, they were packaged and cremated. The entire experimental process of the animals strictly adhered to the regulations for the care and use of laboratory animals as reviewed and approved by the Ethics Committee of The First Affiliated Hospital of Wenzhou Medical University (Wenzhou, China).

### Method validation

We established a stable, rapid, and precise measurement method to simultaneously detect the concentrations of vilazodone and M10 in rat plasma by UPLC-MS/MS, and verified items required by the FDA such as the selectivity, sensitivity, linearity, stability, recovery, precision and accuracy were performed. The method was fully validated according to the guidelines of the China Food and Drug Administration and in compliance with the principles of Guidance for Industry Bioanalytical Method Validation by the FDA [22].

Blank rat plasma, mixed sample of the analytes at lower limit of quantification (LLOQ) concentration and plasma sample of rat orally administered vilazodone were used as analytic specimens to verify the selectivity. Six replicate specimens were used to verify the absence of interferences near the retention times of the targets to check the selectivity of this method. The sensitivity was evaluated by the LLOQ value, and the signal-to-noise ratio was set to be > 10 times. A weighted least squares regression model and the ratio of peak area (analyte/IS) against the nominal concentration of analyte were used to evaluate the calibration curve for the analytes in this study.

The QC samples of vilazodone and its metabolite at four concentrations were used for the estimation of precision and accuracy. The relative error (RE%) was used to assess the precision, and the relative standard deviation (RSD%) was used to assess the accuracy. Inter-day and intra-day precision and accuracy were tested on three consecutive days and on the same day, respectively. Besides, the assessment of the precision and accuracy also needed 1.0 ng/mL (LLOQ) for vilazodone and its metabolite. The six replicate specimens were required in all evaluation. Matrix effects were assessed by comparing the peak areas at respective concentration levels of the post-spiked samples with pure solutions at three concentrations. To estimate extraction recovery, the peak area of vilazodone and M10 was calculated before and after plasma preparation. QC samples at three concentrations were measured in six replicates for the stability assessment, and the stability was separately assessed under four different possible environmental conditions.

### Statistical analysis

The non-compartmental pharmacokinetic analysis in this study was calculated with DAS software (Drug and statistics, Version 2.0, Shanghai University of Traditional Chinese Medicine, China). Origin 9.0 software (Originlab Company, Northampton, MA, USA) was used to draw the plasma concentration-time curves. Statistical Package for the Social Sciences (version 17.0; SPSS Inc., Chicago, IL) was used to perform the unpaired *t*-test analysis, and the criterion for a significant difference was *p* < 0.05.

## Results

### Method development

The MS parameters of vilazodone, M10 and IS were adjusted to get better analytical results. Figure 1 showed the details of mass spectrum of vilazodone, M10 and IS, indicating that the parent ions of vilazodone, M10 and IS were *m/z* 442.12, 443.09, and 434.17, respectively. The most abundant fragment ions belonged to vilazodone, M10 and IS were at *m/z* 197.05, 155.02, 273.06, respectively. Based on these results, the product ions for the three targets of the UPLC-MS/MS method were chosen.

The method we developed in this study had an LLOQ of 1.0 ng/mL for both vilazodone and M10, and the determination was taken to be 2.0 min in total. The methods performed for the determination of vilazodone in plasma were documented [18–20]. However, these bioanalytical methods did not measure the metabolite M10 in biological matrices, so its characteristic had not been characterized in detail. To the best of our knowledge, compared with another study, we had greatly improved the detection accuracy, analytical time and the sensitivity of vilazodone and its metabolite M10 [21].

### Method validation

#### Selectivity

**Fig. 2:**
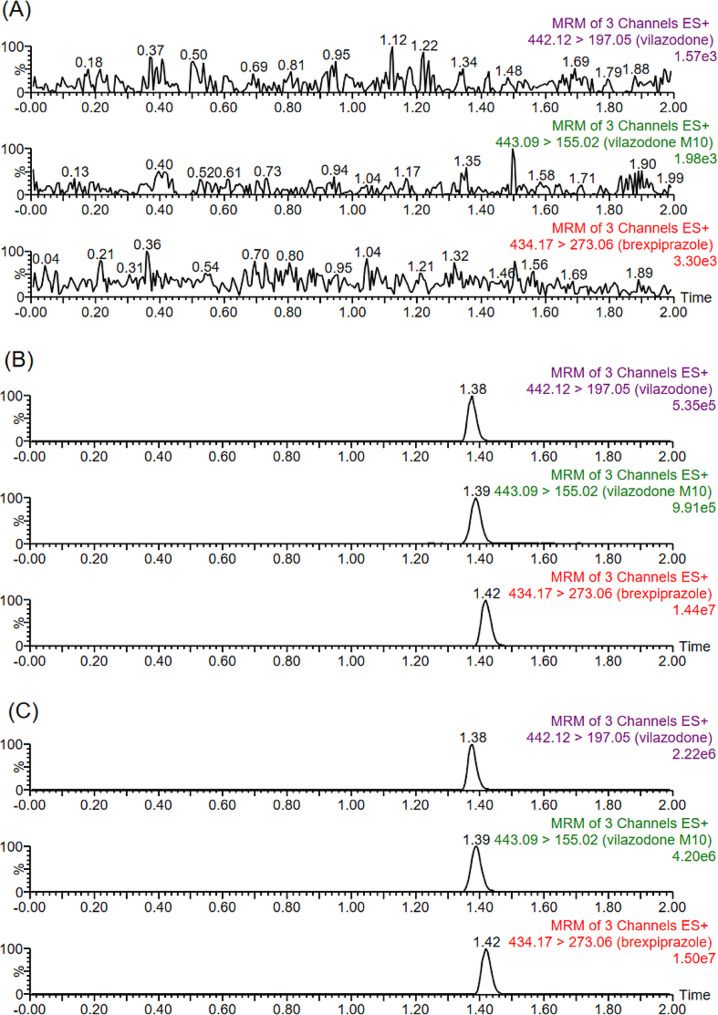
Representative chromatograms of vilazodone, its metabolite and IS in rat plasma. **(A)** blank plasma, **(B)** blank plasma spiked with standard solutions, **(C)** sample from a rat after gavage with 4 mg/kg vilazodone and a subsequent standard plasma preparation

Figure 2 displayed the results of selectivity assessment, which was finished with blank rat plasma, mixed sample of the analytes at LLOQ concentration and plasma sample of rat orally administered vilazodone. It could be seen that no potential endogenous and exogenous interfering substances were found in the respective retention times of vilazodone, M10 and IS, which suggested that the selectivity of this method had been confirmed.

#### Linearity and sensitivity

The calibration curves for vilazodone and its metabolite were constructed using eight different concentrations of the working solutions. The calibration curves were finally calculated as Y = 0.146196 × X + 0.0786286, and Y = 0.336435 × X + 0.161552 for vilazodone and M10, respectively. The values of correlation coefficient were separately 0.9998 and 0.9996, which suggested that this method showed excellent linearities. The LLOQ of both targets were validated with acceptable accuracy and precision, in this experiment, the acceptable LLOQ of both targets were set as 1.0 ng/mL.

#### Accuracy and precision

Table 2 showed the accuracy (RE%) and precision (RSD%) of vilazodone and its metabolite in rat plasma. The accuracy of vilazodone ranged from − 14.9 to 11.2%, and the accuracy of its metabolite ranged from − 10.4 to 13.4%. The highest precision of vilazodone and M10 was 8.9% and 7.3%, respectively. The corresponding lowest precision of vilazodone and M10 was 3.0% and 2.7%, respectively. These results confirmed that the precision and accuracy of this method met FDA standards.

**Table 2:** The precision and accuracy of vilazodone and its metabolite in rat plasma (*n* = 6)

Analytes	Concentration (ng/mL)	Intra-day		Inter-day	
		RSD%	RE%	RSD%	RE%
Vilazodone	1	8.5	-14.9	8.9	-13.5
	2	6.3	3.3	6.7	1.5
	40	4.8	11.2	5.1	10.5
	180	3.0	-4.0	3.6	-4.4
M10	1	7.1	-9.3	7.3	-10.4
	2	5.7	7.5	5.9	4.1
	40	5.2	13.4	5.2	11.9
	180	2.7	-1.4	3.1	-0.8

#### Recovery, matrix effect and stability

We measured the concentrations of vilazodone and its metabolite in rat plasma by this method. Data of the recovery and matrix effect had been shown in Table 3, which suggested that there was no significant matrix effect and splendid recoveries were found in this method. The stability test had been performed under four different possible conditions, including at room temperature for 2 h, at 10 °C for 4 h, three complete freeze-thaw cycles and at -80 °C for 3 weeks. Excellent stability had been found under all four conditions, and the detail data of stability test were listed in Table 4.

**Table 3:** Recovery and matrix effect of vilazodone and its metabolite in rat plasma (*n* = 6)

Analytes	Concentration (ng/mL)	Recovery (%)		Matrix effect (%)	
		Mean ± SD	RSD (%)	Mean ± SD	RSD (%)
Vilazodone	2	89.3 ± 10.6	11.8	100.9 ± 11.0	10.9
	40	93.6 ± 4.6	5.0	100.4 ± 5.1	5.1
	180	94.0 ± 5.1	5.5	102.4 ± 3.4	3.3
M10	2	91.8 ± 4.9	5.4	101.3 ± 6.5	6.4
	40	93.4 ± 3.7	3.9	101.0 ± 5.4	5.3
	180	94.3 ± 6.8	7.2	104.0 ± 3.8	3.7

**Table 4:** Stability results of vilazodone and its metabolite in plasma under different conditions (*n* = 6)

Analytes	Added (ng/mL)	Room temperature 2 h		10 °C, 4 h		Three freeze-thaw		-80 °C, three weeks		
		RSD%	RE%	RSD%	RE%	RSD%	RE%	RSD%	RE%	
Vilazodone	2	8.4	13.4	8.1	10.8	6.4	8.8	7.2	9.4	
	40	6.1	14.6	7.4	13.0	6.3	14.2	5.8	14.4	
	180	2.7	1.8	3.6	1.6	2.4	1.0	4.1	2.0	
M10	2	4.7	14.0	6.6	8.9	4.9	12.2	6.9	11.4	
	40	3.6	14.9	6.1	6.8	3.7	8.4	2.5	9.2	
	180	2.3	4.0	3.1	3.4	1.7	2.5	1.8	1.7	

### Pharmacokinetic study

**Fig. 3:**
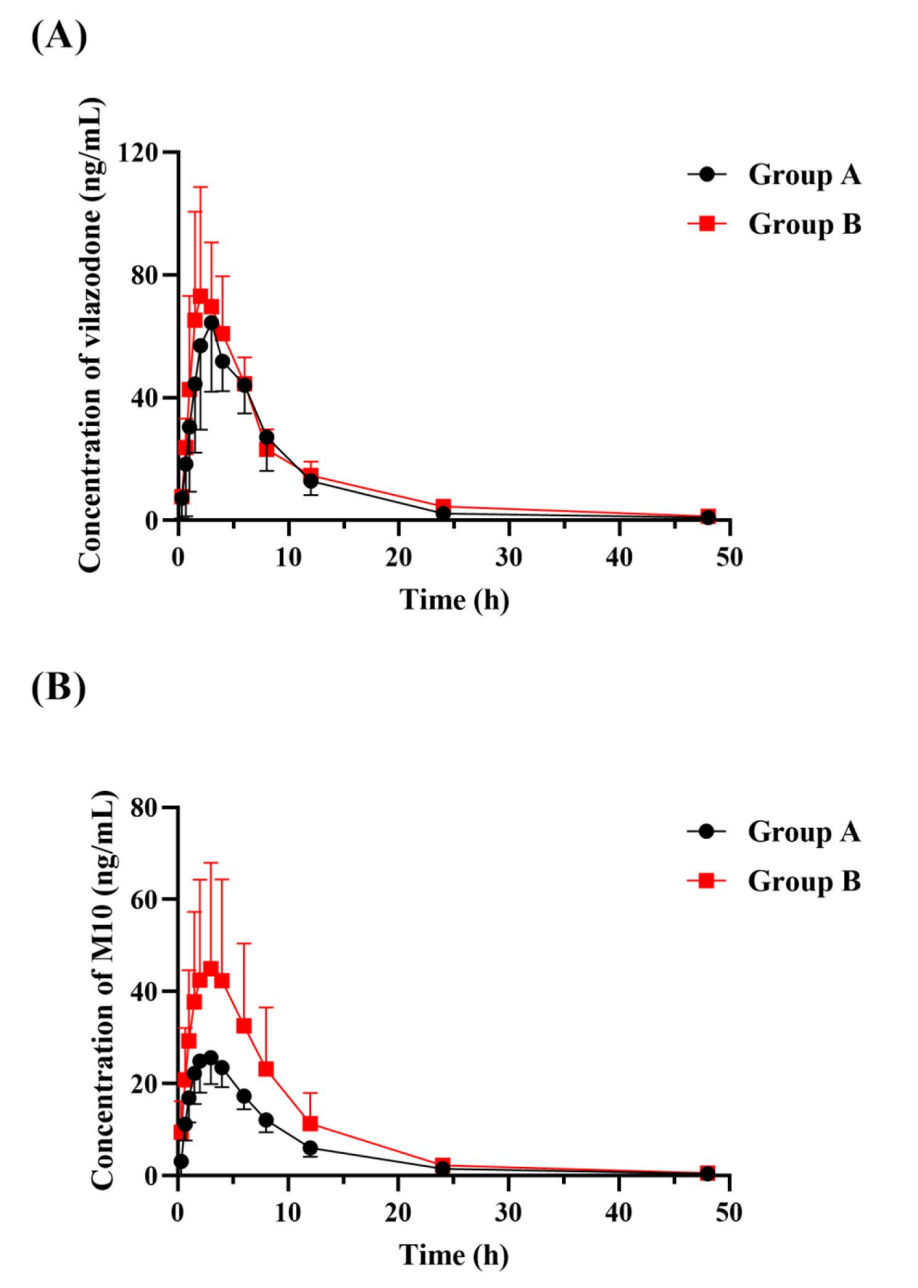
Mean plasma concentration-time curves of vilazodone and its metabolite in rats after oral administration of 4 mg/kg vilazodone with or without 80 mg/kg bergenin (*n* = 4)

The plasma concentration-time curve was shown in Fig. 3, showing the changes in plasma drug concentrations following a single dose 4 mg/kg vilazodone oral administration with or without the combination of 80 mg/kg bergenin. Table 5 showed the detail results of the pharmacokinetic study, and parameters including total clearance (CL), the peak time of plasma concentration (T_max_) and elimination half-time (t_1/2_) had been calculated.

**Table 5:** The main pharmacokinetic parameters of vilazodone and its metabolite in different treatment groups of rats. Group A: the control group; Group B: 80 mg/kg bergenin. (*n* = 4, Mean ± SD)

Parameters	Vilazodone		M10	
	Group A	Group B	Group A	Group B
AUC_0→t_ (ng/mL•h)	552.84 ± 150.37	651.32 ± 181.53	254.34 ± 25.17	454.05 ± 243.44
AUC_0→∞_ (ng/mL•h)	553.16 ± 150.56	660.67 ± 178.73	255.91 ± 23.88	455.61 ± 243.35
t_1/2_ (h)	4.32 ± 0.23	8.41 ± 2.41*	5.99 ± 1.71	5.23 ± 1.54
T_max_ (h)	3.00 ± 0.82	2.75 ± 0.96	3.00 ± 0.82	2.75 ± 0.50
CLz/F (L/h)	7.61 ± 1.90	6.37 ± 1.59	15.73 ± 1.45	10.31 ± 3.92*
C_max_ (ng/mL)	69.37 ± 21.33	81.86 ± 28.68	26.19 ± 5.90	45.30 ± 23.07

Compared with Group A, *P < 0.05

### Pharmacokinetics of vilazodone and its metabolite in rats

The results indicated that vilazodone was rapidly absorbed and metabolized to M10 after oral administration of 4 mg/kg vilazodone alone. The peak concentrations (C_max_) of vilazodone and M10 were 69.37 ± 21.33 and 26.19 ± 5.90 ng/mL, respectively. Correspondingly, the T_max_ of both vilazodone and M10 were 3.00 ± 0.82 h. In addition, vilazodone showed smaller t_1/2_ and CLz/F than M10, with values of 4.32 ± 0.23 h and 7.61 ± 1.90 L/h, respectively. For M10, the results were 5.99 ± 1.71 h and 15.73 ± 1.45 L/h, respectively.

### Effect of bergenin on the pharmacokinetics of vilazodone and its metabolite

After the combination of 80 mg/kg bergenin in rats, the t_1/2_ of vilazodone was significantly prolonged by 94.7%. The CLz/F of M10 was decreased by 34.5% after combined with bergenin. Interestingly, with the addition of bergenin, the AUC and C_max_ of vilazodone and its metabolite were improved compared to those without bergenin, however, these differences were not statistically significant.

## Discussion

Plants of Genus Bergenia has been used as a traditional Chinese medicine for thousands of years, bergenin is one of its main active ingredients [1, 2]. Except for the treatment of various diseases [9, 10], bergenin is also commonly found in some cosmetics in daily life. Because of the various pharmacological effects and the daily application of bergenin, there is a possibility of simultaneous intake of bergenin and vilazodone. Therefore, the potential drug-drug interaction between bergenin and vilazodone needs to be explored.

In this study, we observed that in combination with bergenin, the t_1/2_ of vilazodone was significantly prolonged and the CLz/F of M10 was decreased. It suggested that begernin inhibited the metabolism of vilazodone in rats. Previous studies showed that bergenin is a natural a PPARγ agonist and a human liver CYP3A4 and CYP2C9 inhibitor [11]. Vilazodone, commonly used to treat major depressive disorder in adults, is metabolized mainly by the hepatic CYP3A4 enzyme system and one of the major inactive metabolites of vilazodone is M10 [12, 13, 17]. These findings are consistent with our observations, suggesting that the inhibition of CYP3A4 enzyme system may be the underlying mechanism for the drug-drug interaction between bergenin and vilazodone. Boinpally et al. investigated the pharmacokinetics of vilazodone when co-administered with ketoconazole (CYP3A4 inhibitor) to assess the effect of CYP450 inhibitors on the pharmacokinetics of vilazodone, which supports our hypothesis [23].

## Conclusions

In conclusion, we firstly established and verified an analysis method of vilazodone and its metabolite M10 in rat plasma. We also used this method to explore the pharmacokinetics of vilazodone and its drug-drug interaction with bergenin in this study. Results of our research indicated that the metabolism of vilazodone in rats can be significantly affected by bergenin. In clinical application, we should pay attention to the dose of vilazodone in combination with bergenin. In addition, the pharmacokinetic of vilazodone when co-administered with other drugs that can affect CYP450 activity requires further study to ensure the safety of vilazodone in clinical application.

## Data Availability

The raw data supporting the conclusion of this article will be made available by the authors, without undue reservation.
